# IL-17 promotes IL-18 production via the MEK/ERK/miR-4492 axis in osteoarthritis synovial fibroblasts

**DOI:** 10.18632/aging.205462

**Published:** 2024-01-22

**Authors:** Kun-Tsan Lee, Chih-Yang Lin, Shan-Chi Liu, Xiu-Yuan He, Chun-Hao Tsai, Chih-Yuan Ko, Yuan-Hsin Tsai, Chia-Chia Chao, Po-Chun Chen, Chih-Hsin Tang

**Affiliations:** 1 Department of Post-Baccalaureate Medicine, National Chung-Hsing University, Taichung, Taiwan; 2 Department of Orthopedics, Taichung Veterans General Hospital, Taichung, Taiwan; 3 Translational Medicine Center, Shin-Kong Wu Ho-Su Memorial Hospital, Taipei City, Taiwan; 4 Institute of Biomedical Sciences, Mackay Medical College, New Taipei City, Taiwan; 5 Department of Pharmacology, School of Medicine, China Medical University, Taichung, Taiwan; 6 Department of Orthopedic Surgery, China Medical University Hospital, Taichung, Taiwan; 7 Department of Sports Medicine, College of Health Care, China Medical University, Taichung, Taiwan; 8 Graduate Institute of Biomedical Science, China Medical University, Taichung, Taiwan; 9 Department of Orthopedics, Show-Chwan Memorial Hospital, Changhua, Taiwan; 10 Department of Respiratory Therapy, Fu-Jen Catholic University, New Taipei City, Taiwan; 11 School of Life Science, National Taiwan Normal University, Taipei, Taiwan; 12 Chinese Medicine Research Center, China Medical University, Taichung, Taiwan; 13 Department of Medical Laboratory Science and Biotechnology, College of Health Science, Asia University, Taichung, Taiwan; 14 Department of Medical Research, China Medical University Hsinchu Hospital, Hsinchu, Taiwan

**Keywords:** osteoarthritis, IL-17, IL-18

## Abstract

The concept of osteoarthritis (OA) as a low-grade inflammatory joint disorder has been widely accepted. Many inflammatory mediators are implicated in the pathogenesis of OA. Interleukin (IL)-18 is a pleiotropic cytokine with versatile cellular functions that are pathogenetically important in immune responses, as well as autoimmune, inflammatory, and infectious diseases. IL-17, a proinflammatory cytokine mainly secreted by Th17 cells, is upregulated in OA patients. However, the role of IL-17 in OA progression is unclear. The synovial tissues collected from healthy donors and OA patients were used to detect the expression level of IL-18 by IHC stain. The OA synovial fibroblasts (OASFs) were incubated with recombinant IL-17 and subjected to Western blot, qPCR, and ELISA to examine IL-18 expression level. The chemical inhibitors and siRNAs which targeted signal pathways were used to investigate signal pathways involved in IL-17-induced IL-18 expression. The microRNAs which participated IL-18 expression were surveyed with online databases miRWalk and miRDB, followed by validation with qPCR. This study revealed significantly higher levels of IL-18 expression in synovial tissue from OA patients compared with healthy controls, as well as increased IL-18 expression in OASFs from rats with severe OA. *In vitro* findings indicated that IL-17 dose-dependently promoted IL-18 production in OASFs. Molecular investigations revealed that the MEK/ERK/miR-4492 axis stimulated IL-18 production when OASFs were treated with IL-17. This study provides novel insights into the role of IL-17 in the pathogenesis of OA, which may help to inform OA treatment in the future.

## INTRODUCTION

Osteoarthritis (OA), a degenerative joint disease that mainly affects elderly people [1], gradually breaks down joint cartilage and causes changes in the underlying bone, most frequently in the hands, knees, hips and spine [2]. Subchondral bone sclerosis and cartilage degeneration associated with OA have been well characterized [3]. OA risk factors include joint injury, obesity, aging, and genetics [4, 5]. Synovium-secreted factors related to OA progression mostly involve matrix metalloproteinases (MMPs), interleukin-1β (IL-1β), IL-6, IL-15, IL-17, and CCL2 [6]. Elucidation of the molecular mechanisms and main factors involved in OA pathogenesis may help with the development of novel therapeutic targets that relieve OA pain or prevent the disease from progressing.

IL-18, a member of IL-1 family, is secreted by T cells and stimulates interferon-γ (IFN-γ) production [7]. IL-18 is initially secreted as an inactive precursor that is enzymatically cleaved by inflammasomes into a bioactive cytokine [8]. IL-18 in the joint is released by chondrocytes [9], osteoblasts [10], and fibroblast-like synoviocytes [11]. Increased levels of IL-18 expression in synovial fluid, synovium, cartilage, and blood serum are positively correlated with OA progression [12–15]. In OA chondrocytes, IL-18 upregulates levels of inducible nitric oxide synthase (iNOS), cyclooxygenase-2 (COX-2) and IL-6 expression, leading to the inflammatory responses of chondrocytes [9]. IL-18 also upregulates MMP expression in OA chondrocytes [16] and decreases the production of proteoglycans, aggrecan, and type II collagen [17, 18], leading to the loss of articular cartilage. Thus, IL-18 plays a key role in OA progression.

The inflammation-induced release of IL-17 is produced by innate immune cell populations consisting of T helper 17 (Th17), mast, and myeloid cells [19], and underlies the progression of various autoimmune diseases such as rheumatoid arthritis (RA), multiple sclerosis, and inflammatory bowel disease [20]. Increasing evidence suggests that IL-17 is a key mediator in OA pathogenesis. Previous reports have found higher levels of IL-17 in serum and synovial fluid from OA patients than from healthy donors, and increased IL-17 levels are positively correlated with OA severity scores [21–24]. Furthermore, IL-17 is reported to induce expression of inflammatory mediators such as IL-6, IL-8, C-C motif chemokine ligand 2 (CCL2), C-X-C motif chemokine ligand 1 (CXCL1), COX-2, and iNOS in OA cells [11, 25]. However, the role of IL-17 in OA progression remains unclear.

Numerous studies have indicated a positive correlation between the expression of IL-17 and IL-18 in various disease statuses [26–28]. However, the regulatory mechanism between IL-17 and IL-18 has been poorly discussed. In this study, we found that IL-17 promotes IL-18 expression in OA synovial fibroblasts (OASFs) via a MEK/ERK-dependent molecular mechanism. The microRNA 4492 (miR-4492) also participated in the promotion of IL-18 expression in OASFs after IL-17 treatment. In summary, the MEK/ERK/miR-4492 axis is responsible for the promotion of IL-18 expression when OASFs are treated with IL-17. Our findings provide novel insights into the role of IL-17 in OA pathogenesis.

## MATERIALS AND METHODS

### Materials

All cell culture materials, including Dulbecco’s Modified Eagle Medium (DMEM), fetal bovine serum (FBS), trypsin, antibiotics penicillin-streptomycin (10,000 U/mL), and Lipofectamine 2000 were obtained from Invitrogen (Carlsbad, CA, USA). Plastic dishes and plates for cell culture and sample collection were purchased from Corning Inc. (Corning, NY, USA). The polyvinylidene difluoride (PVDF) membrane and Immobilon™ Western Chemiluminescent HRP Substrate were purchased from Millipore (Billerica, MA, USA). Detailed antibody information is as follows: IL-18 (Cat. No. GTX101368; GeneTex; Hsinchu City, Taiwan), p-MEK (Cat. No. 2338S; Cell Signaling Technology; Danvers, MA, USA), p-ERK (Cat. No. SC-7383; Santa Cruz Biotechnology; Dallas, TX, USA), MEK (Cat. No. SC-6250; Santa Cruz Biotechnology), ERK (Cat. No. SC-1647; Santa Cruz Biotechnology), and β-actin (Cat. No. SC-47778; Santa Cruz Biotechnology). All chemical inhibitors were obtained from Sigma-Aldrich (St. Louis, MO, USA), including PD98059, U0126, and ERKII. IL-17 recombinant protein was purchased from PeproTech (Rocky Hill, NJ, USA). Small interfering RNAs (siRNAs) specific for IL-18, MEK, and ERK were purchased from Dharmacon™ (Lafayette, CO, USA). The microRNA mimic specific for miR-4492 was purchased from Thermo Fisher Scientific Inc. (Waltham, MA, USA).

### Bioinformatic analysis

For bioinformatic analysis, levels of mRNA expression were retrieved from the Gene Expression Omnibus (GEO) database (accession code: GSE89408). Levels of mRNA expression were determined by reads per expectation maximization (RSEM).

### Cell culture

Fresh tissue samples of OA synovium were obtained from OA patients undergoing knee arthroplasty. Normal synovium samples were obtained from patients undergoing arthroscopic surgery for knee injury or internal joint derangement. The study was approved by China Medical University Hospital and written informed consent was obtained from all study participants. The synovium was cut into 1–2 mm-sized pieces then digested with collagenase (1 mg/mL) in DMEM medium for 2 h at 37° C. Dissociated cells were collected and kept as previously described [29–31]. OASFs were kept in DMEM supplied with 10% FBS and antibiotics penicillin-streptomycin to make a complete medium. The cells were maintained in an incubator with a humidified incubator at 37° C under 5% CO_2_. All cells used in the experiments of this study were obtained from the 6th to 8th passages.

### RNA extraction and quantitative real-time PCR

Total RNA was extracted from the cells using an easy-BLUE™ Total RNA Extraction Kit (iNTRON Biotechnology, Seoul, Korea), according to the manufacturer’s instructions. One μg total RNA was subjected to a reverse transcription reaction to generate complementary DNA (cDNA) by reverse transcriptase (Invitrogen, Carlsbad, CA, USA). A quantitative real-time PCR (qPCR) reaction was conducted with the StepOnePlus™ machine (Applied Biosystems, Foster City, CA, USA) using SYBR Green qPCR Master Mix (KAPA Biosystem, Woburn, MA, USA), according to the manufacturer’s protocol. Reaction conditions for qPCR were as follows: 10 min at 95° C; 40 cycles of 15 s at 95° C and 60 s at 60° C. All primers were designed by and purchased from Sigma-Aldrich (St. Louis, MO, USA). GAPDH expression was used as internal control for normalization. The cycle threshold (Ct) was set above the non-template control background and within the linear phase of target gene amplification for calculating the threshold cycle numbers at which the transcript was detected. All study data are representative from 3 independent experiments.

### Western blot analysis

Western blot was conducted as our previous study [32]. Total cell lysates were collected with RIPA buffer. Proteins were resolved on SDS-PAGE and transferred to PVDF membranes. Blots were blocked with 5% nonfat milk for 1 h at room temperature, then incubated with primary antibodies against IL-18, p-MEK, MEK, p-ERK, and ERK (1:1000) for 1 h at room temperature. After 3 washes with PBS, the blots were incubated with horseradish peroxidase (HRP)-linked secondary antibody (1:5000) for 1 h at room temperature. The blots were then visualized using a ImageQuant LAS 4000 camera (GE Healthcare, Little Chalfont, UK). Quantitative data were obtained using ImageJ software (National Institutes of Health, Bethesda, MD, USA).

### Anterior cruciate ligament transection (ACLT) animal model

All animal procedures were approved by the Institutional Animal Care and Use Committee (IACUC) of China Medical University Hospital. ACLT animal model was conducted as previous report [33]. Male Sprague-Dawley (SD) rats (8 weeks old) were used in the study experiment. Briefly, the left knee was operated on under surgically sterile conditions. After anesthetizing the rats with inhaled isoflurane, the joint capsules were opened and the ACL fibers were transected with a scalpel, then the entire medial meniscus was removed by medial parapatellar mini-arthrotomy. After surgery, the joints were washed with sterile PBS and sutured, followed by 1 week of antibiotic treatment (ampicillin; 50 mg/kg). Sham-operated rats served as controls (these rats underwent the same surgery without ACL transection). All rats were allowed to move freely in plastic cages until necropsy at 8 weeks post-surgery. Rat knee joints were excised promptly after sacrifice and fixed in 3.7% formaldehyde, decalcified in 10% EDTA, then dehydrated in ethanol/xylene. The tissues were paraffin-embedded and cut into 5-μm thick sections. The sections were stained with hematoxylin and eosin (H&E) to confirm histological features of knee joints; Safranin O/fast green was used to evaluate cartilage degradation. Briefly, the sections from all experiment groups were stained with Safranin O/fast green or H&E and examined under a light microscope for histopathological changes. Cartilage destruction was evaluated by the Osteoarthritis Research Society International (OARSI) score system established by the International Association for Osteoarthritis Research [34]. The OARSI score system includes 6 grades (Grade 0 = no cartilage degeneration; Grade 1 = Minimal degeneration, 5–10% of the total projected cartilage area affected by matrix or chondrocyte loss; Grade 2 = Mild degeneration, 11–25% affected; Grade 3 = Moderate degeneration, 26–50% affected; Grade 4 = Marked degeneration, 51–75% affected; Grade 5 = Severe degeneration, greater than 75% affected). The scoring was evaluated in a blind fashion by two independent individuals and the scores were averaged to minimize observer bias. Levels of IL-18 expression were detected by immunohistochemistry (IHC) staining.

### IHC staining

IHC staining was performed as previous study [35]. The fresh synovium samples from OA patients and healthy donors were fixed in 1% formaldehyde, decalcified in 10% EDTA, then dehydrated in ethanol/xylene. The tissues were paraffin-embedded and cut into 5-μm thick sections. The sections were stained with H&E, then incubated with anti-IL-18 primary antibody (1:100) for 1 h at room temperature. The detection of IL-18 antibody in sections was conducted using a NovoLink Polymer Detection Systems kit (Leica Biosystems, Wetzlar, Germany) according to the manufacturer’s protocol. Levels of IL-18 expression are represented by the percentage of stained area out of the total area in each microscopy photo of the slides. Three random fields taken from each slide were used for quantification.

### Transfection of miRNA mimic

Cells were transfected with 25 nM siRNAs or miRNA mimic using Lipofectamine 2000 (Invitrogen Life Technology, Waltham, MA, USA), in accordance with the manufacturer’s instructions. After 24 hours post-transfection, the cells were stimulated by IL-17 and subjected to qPCR and Western blot assays as described in Figure Legends section.

### ELISA assay

The quantification of IL-18 expression in the cell culture media was performed using an ELISA kit from R&D Systems, Inc. (Minneapolis, MN, USA). After subjecting OASFs to 24 hours of IL-17 treatment in the presence of specified inhibitors, siRNAs, or miRNA mimics, the cell culture media were collected and assessed for IL-18 secretion utilizing the ELISA kit, in accordance with the manufacturer’s instructions.

### Statistical analysis

All reported values are means ± standard deviations (S.D.) of independent experiments. Statistical analysis between two samples was performed using the Student’s *t*-test. Statistical comparisons involving more than two groups were performed using one-way analysis of variance (ANOVA) with the Fisher’s Least Significant Difference (LSD) *post-hoc* test. In all cases, *P* < 0.05 was considered significant.

### Availability of data and materials

The data sets used and analyzed during the current study are available from the corresponding author on reasonable request.

## RESULTS

### IL-18 was upregulated in synovium tissues during OA progression

**Figure 1:**
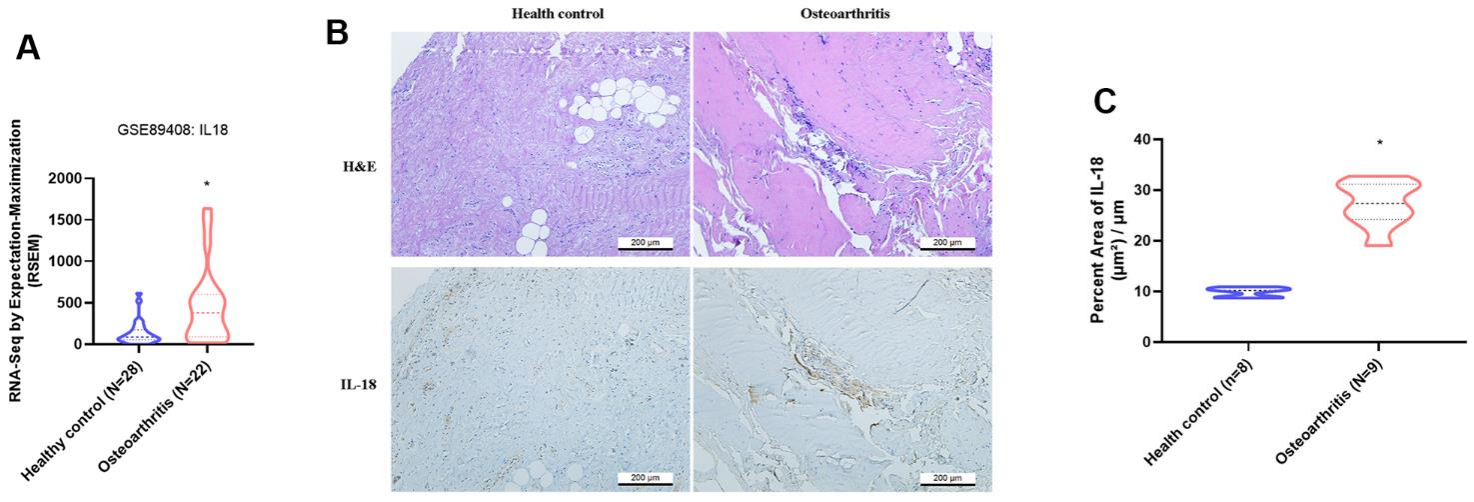
**IL-18 is upregulated in clinical synovial tissue from OA patients.** (**A**) Patterns of mRNA expression in the GEO dataset GSE890408 in synovial tissue samples from OA patients and normal healthy donors were used to analyze IL-18 expression. (**B**) Synovial tissues from the study cohorts of OA patients and normal healthy donors were subjected to H&E and IHC staining for detecting IL-18 expression. (**C**) Quantification of IHC results from Figure 1B. Results are expressed as the means ± S.D. *p<0.05 compared with healthy controls.

**Figure 2:**
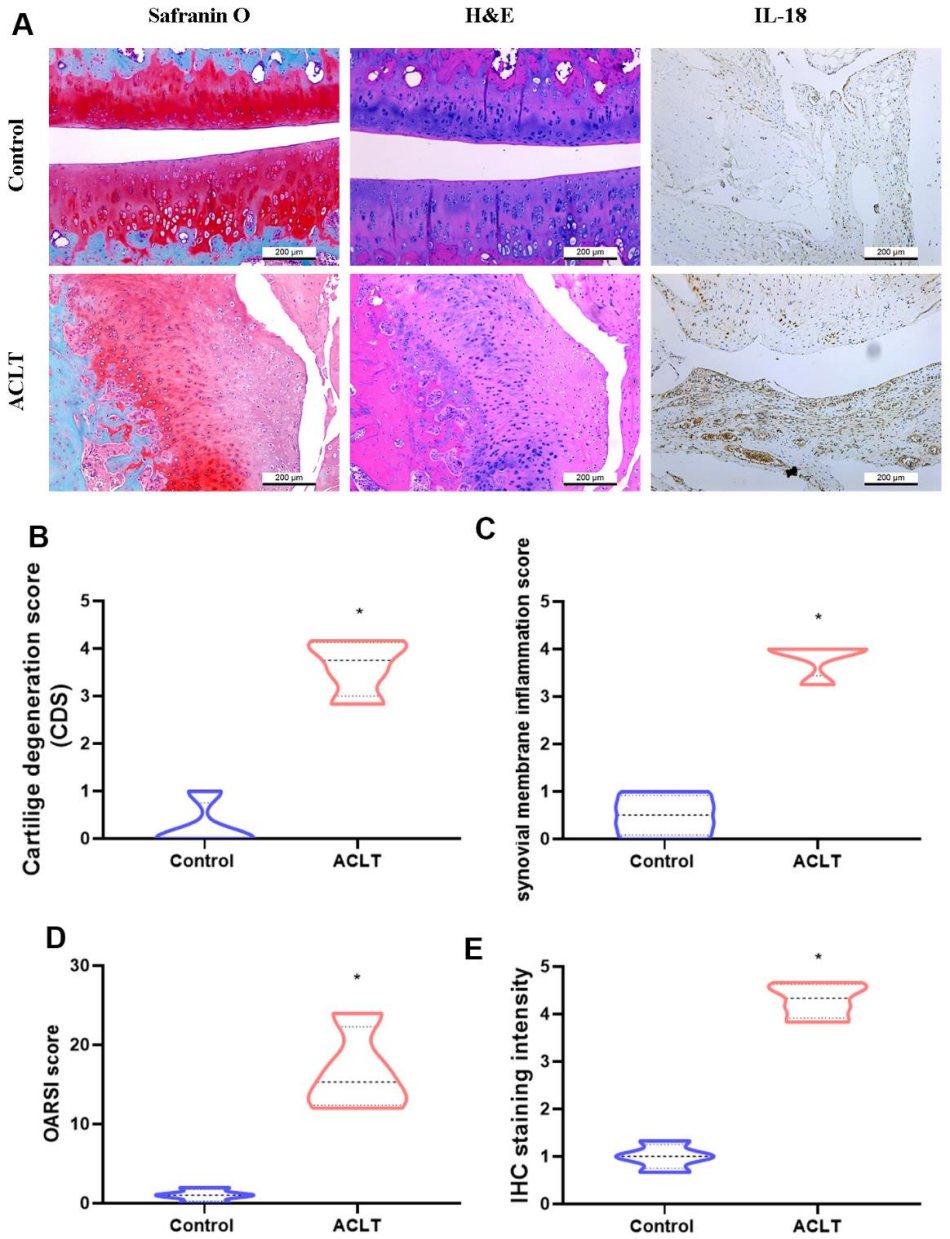
**Histologic assessments revealed increases in IL-18 expression in ACLT rats.** (**A**) All rats were sacrificed at 8 weeks after surgery. Paraffin-embedded sections of knee joints were subjected to Safranin O/fast green, H&E, and IHC staining for analyses of IL-18 expression. (**B**–**D**) The severity of knee OA was assessed by cartilage degeneration scores (CDS), synovial membrane inflammation scores, and Osteoarthritis Research Society International (OARSI) scores. (**E**) Levels of IL-18 expression were evaluated by the intensity of IHC staining. Results are expressed as the means ± S.D. *p<0.05 compared with controls.

The GEO dataset contained RNA-seq results of synovial tissue from OA patients and healthy donors, and showed that IL-18 was significantly upregulated in OA synovium tissue compared with healthy donor tissue (Figure 1A). Levels of IL-1β, a key player in OA pathogenesis [36], were also significantly higher in OA patients compared with healthy donors (Supplementary Figure 1). IHC staining revealed upregulated IL-18 expression in synovial specimens from OA patients compared with healthy donor specimens (Figure 1B, 1C). H&E and IHC staining identified increased levels of IL-18 expression in synovium from ACLT rats compared with controls (Figure 2). It appears that IL-18 is upregulated during OA progression and may promote the disease progression.

### IL-17 induced IL-18 expression in OASFs

**Figure 3:**
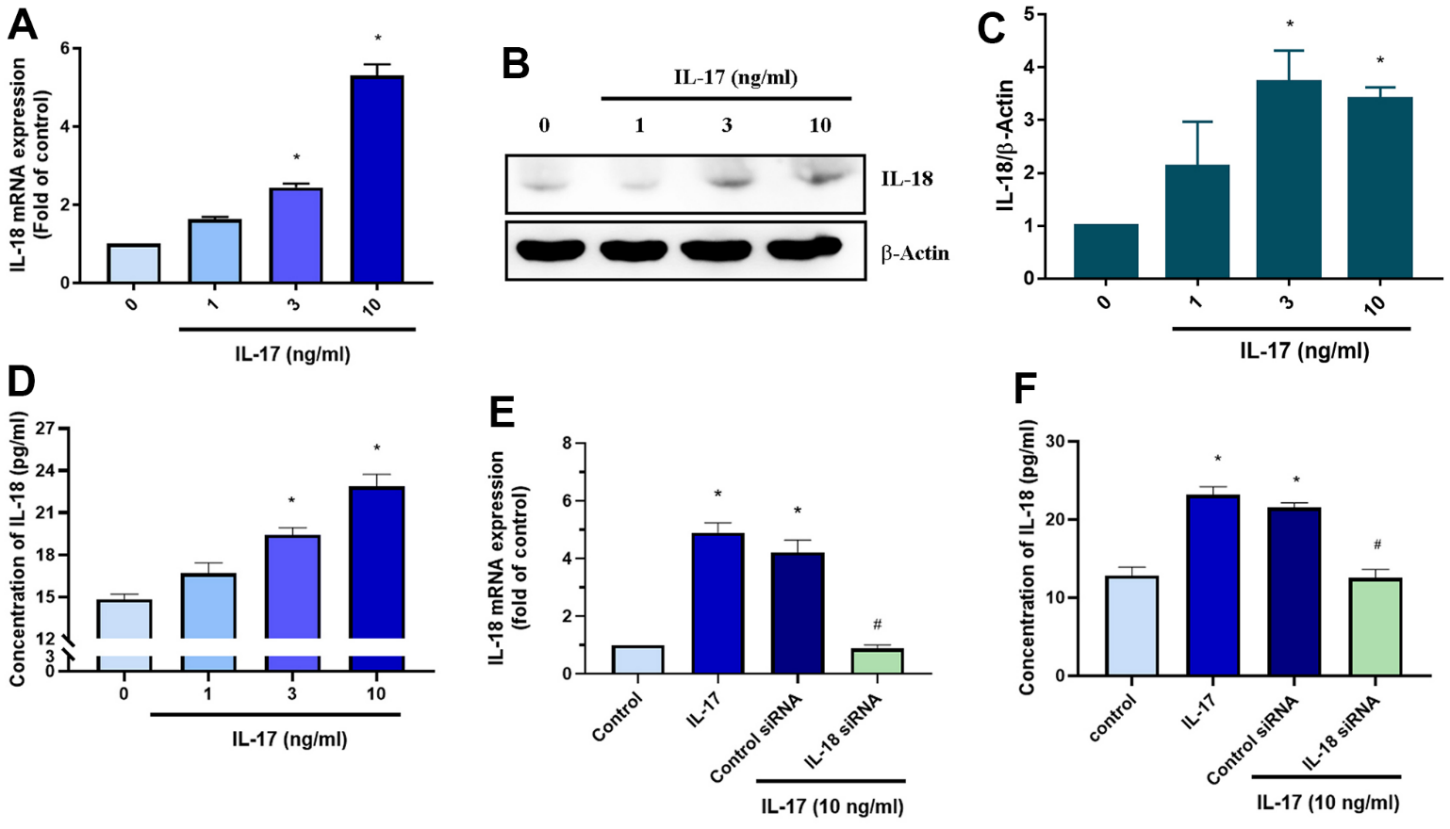
**IL-17 promotes the production of IL-18 in OASFs.** (**A**–**D**) OASFs were incubated with different concentrations of IL-17 (0–10 ng/mL) for 24 h. Levels of IL-18 expression were detected by qPCR (**A**), Western blot (**B**, **C**), and ELISA (**D**) analyses. (**E**, **F**) OASFs were transfected with IL-18 siRNA then incubated with IL-17 (10 ng/mL) for 24 h and assessed for IL-18 expression. Results are expressed as the means ± S.D. *p<0.05 compared with controls.

We next sought to determine what molecular mechanism is involved in IL-18 upregulation during OA progression. Interestingly, markedly higher numbers of circulating Th17 cells, responsible for IL-17 secretion, have been observed in patients with OA than in healthy donors [37]. We therefore examined whether IL-17 promotes IL-18 expression in OASFs. As shown in Figures 3A–3C, qPCR and Western blot analyses revealed that IL-17 dose-dependently promoted IL-18 expression in OASFs. IL-18 secretion was also increased in response to IL-17 stimulation in OASFs (Figure 3D). Finally, transfection of IL-18 siRNA significantly abrogated IL-18 expression after IL-17 treatment, confirming involvement of the IL-17/IL-18 axis in OASFs (Figure 3E, 3F). These data demonstrate that IL-17 significantly increases IL-18 production in OASFs.

### IL-17-induced promotion of IL-18 production involves MEK signaling

**Figure 4:**
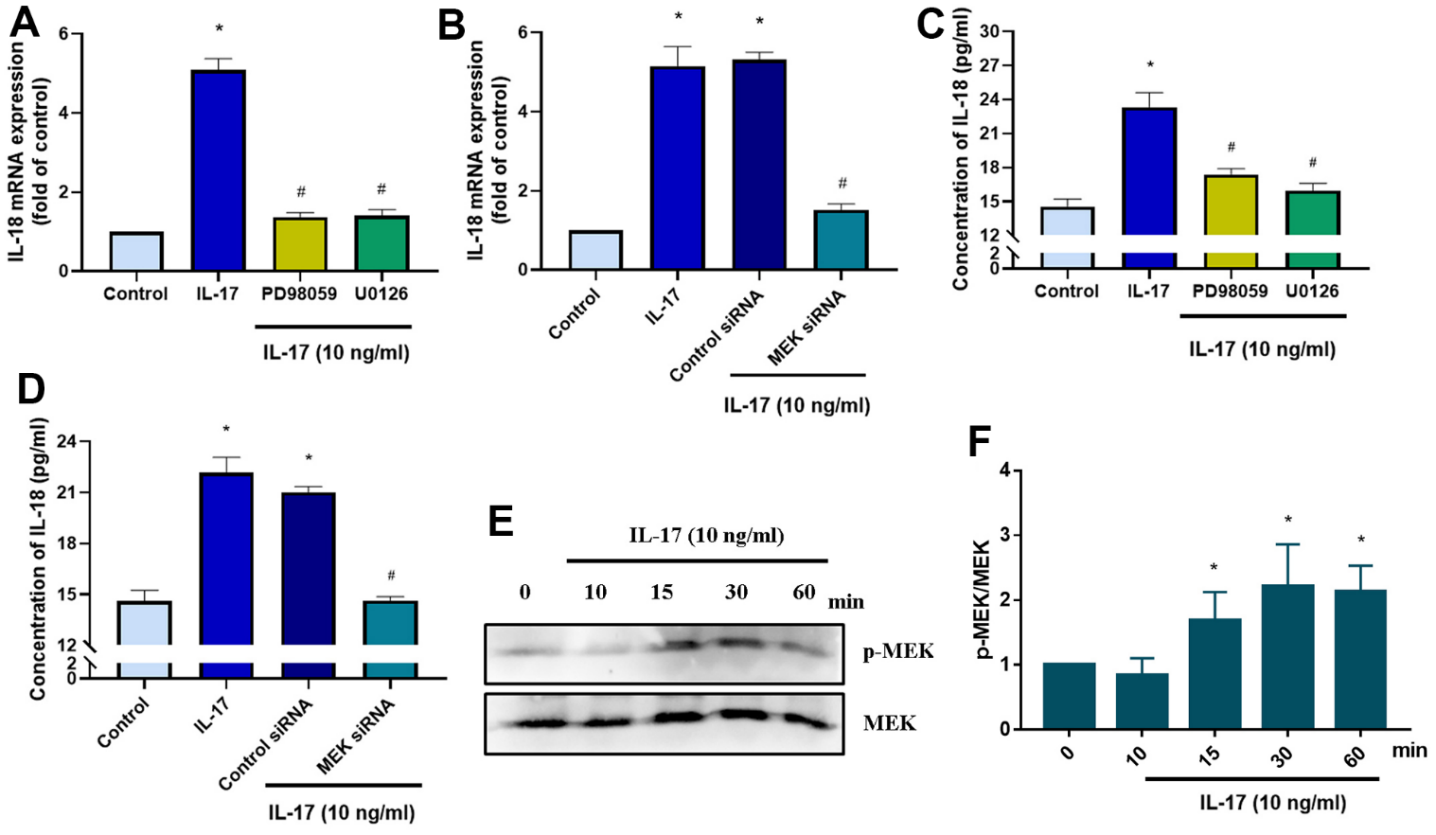
**IL-17-induced promotion of IL-18 production in OASFs requires MEK signaling.** (**A**) OASFs were pretreated with MEK inhibitors PD98059 (10 μM) or U0126 (5 μM) for 1 h, then incubated with IL-17 (10 ng/mL) for 24 h. IL-18 expression was determined by qPCR. (**B**) OASFs were transfected with MEK siRNA or control siRNA for 24 h, then stimulated with IL-17 (10 ng/mL) for a further 24 h. IL-18 expression was assessed by qPCR. (**C**, **D**) OASFs were treated as described in Figure 4A, 4B. IL-18 production was examined by ELISA. (**E**) OASFs were stimulated with IL-17 (10 ng/mL) for different time intervals (0–60 min). The total cell lysates were collected and Western blot assessed MEK protein phosphorylation. (**F**) The quantification result of MEK protein phosphorylation was shown. Results are expressed as the means ± S.D. * p<0.05 compared with controls; # p<0.05 compared with the IL-17-treated group.

**Figure 5:**
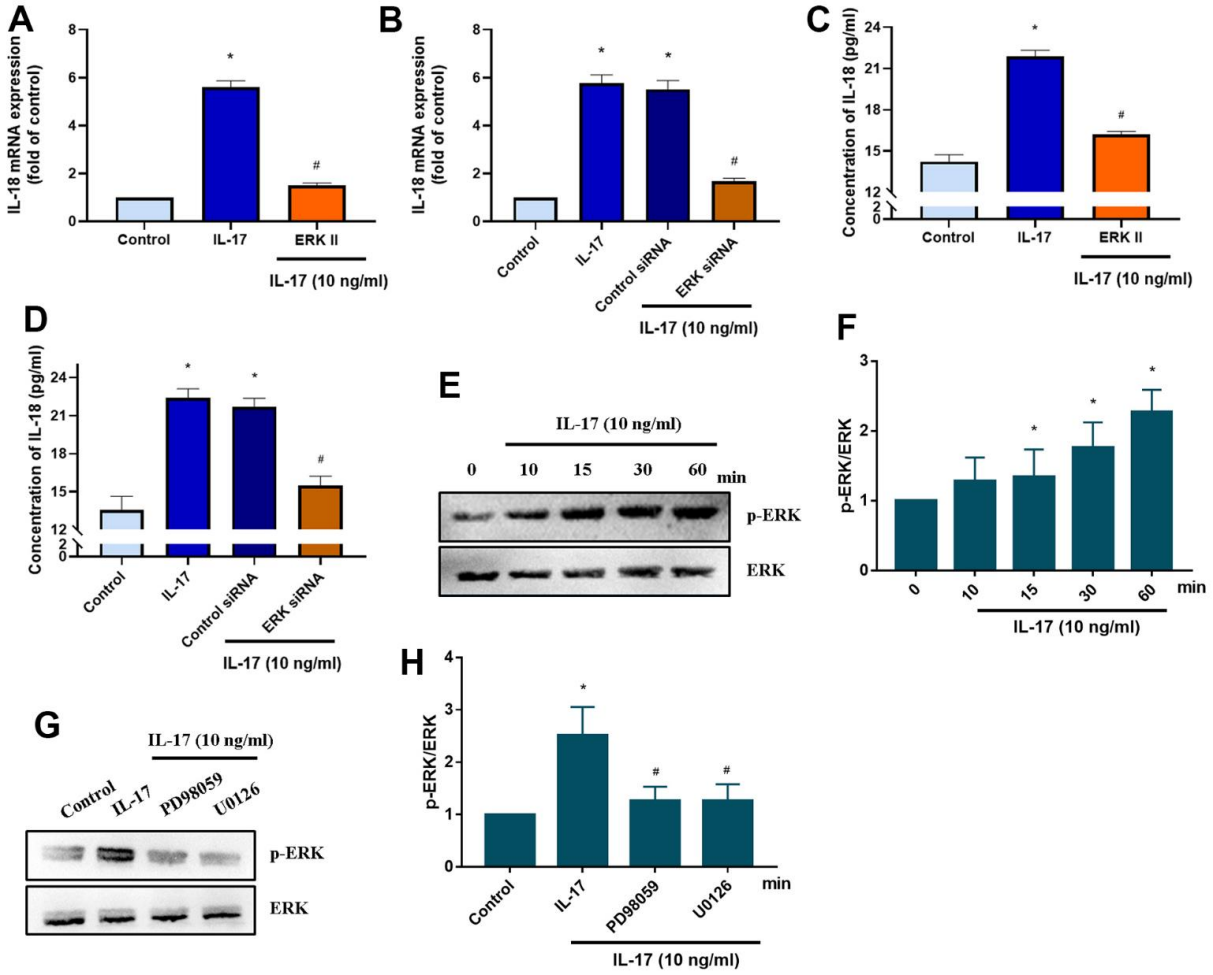
**ERK signaling mediates IL-18 production in OASFs in response to IL-17 stimulation.** (**A**) OASFs were pretreated with the ERK inhibitor ERKII (5 μM) for 1 h, then incubated with IL-17 (10 ng/mL) for 24 h. IL-18 expression was determined by qPCR. (**B**) OASFs were transfected with ERK siRNA or control siRNA for 24 h, then stimulated with IL-17 (10 ng/mL) for a further 24 h. IL-18 expression was assessed by qPCR. (**C**, **D**) OASFs were treated as described in Figure 5A, 5B. IL-18 production was examined by ELISA. (**E**, **F**) OASFs were stimulated with IL-17 (10 ng/mL) for different time intervals (0–60 min). The total cell lysates were collected and Western blot assessed ERK protein phosphorylation. The quantification of blot was shown in Figure 5F. (**G**, **H**) OASFs were pretreated with PD98059 (10 μM) or U0126 (5 μM) for 1 h, then stimulated with IL-17 (10 ng/mL) for 60 min. The total cell lysates were collected and Western blot assessed ERK protein phosphorylation. ERK protein was used as the internal control. The quantification of blot was shown in Figure 5H. Results are expressed as the means ± S.D. *p<0.05 compared with controls; #p<0.05 compared with the IL-17-treated group.

The MEK signaling pathway is activated by the binding of the IL-17 family to its receptor [38]. We therefore investigated whether IL-17-induced stimulation of IL-18 expression involves MEK signaling. Pretreatment with MEK specific inhibitors (PD98095 and U0126) significantly downregulated IL-18 expression in OASFs in response to IL-17 stimulation (Figure 4A), while MEK siRNA transfection of OASFs significantly inhibited IL-17-induced stimulation of IL-18 expression (Figure 4B). Pretreating OASFs with PD98095 and U0126 also blocked IL-18 secretion in OASFs, indicating the role of MEK signaling in the mediation of IL-18 production in response to IL-17 treatment (Figure 4C, 4D). Finally, treatment of OASFs with IL-17 dramatically enhanced MEK phosphorylation (Figure 4E, 4F). ERK signaling is activated by its upstream regulator MEK, and the MEK/ERK cascade is responsible for a variety of cellular processes including the proliferation, differentiation, and development of cells [39]. After OASFs were treated with IL-17, blocking of ERK signaling transduction with the ERK inhibitor (ERK II) or siRNA significantly decreased IL-18 expression (Figure 5A, 5B). IL-17-induced secretion of IL-18 was also abolished by the ERK inhibitor and siRNA (Figure 5C, 5D). An analysis of IL-17 phosphorylation revealed that ERK was time-dependently activated by exposure to IL-17 (Figure 5E, 5F). Pretreatment of OASFs with MEK specific inhibitors (PD98059 and U0126) markedly reversed ERK phosphorylation induced by IL-17 treatment (Figure 5G, 5H). These findings suggest that MEK/ERK activation is required for IL-18 production in OASFs after IL-17 treatment.

### Downregulation of miR-4492 contributes to IL-18 expression in OASFs

**Figure 6:**
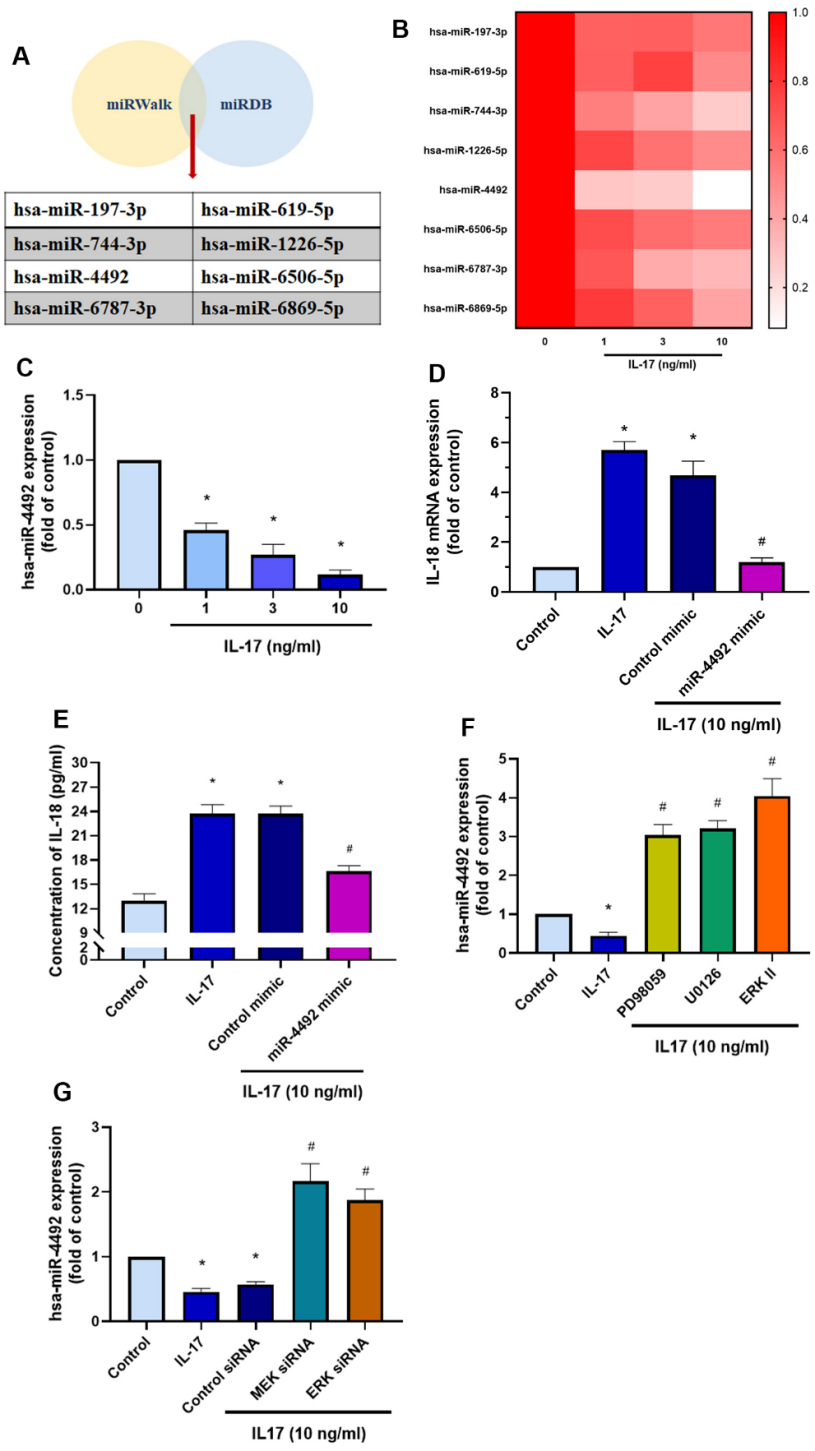
**The MEK/ERK/miR-4492 axis contributes to IL-18 production in OASFs in response to IL-17 stimulation.** (**A**) Online databases for miRNA target prediction (miRWalk and miRDB) were used to screen for candidate miRNAs that potentially target IL-18 mRNA. (**B**, **C**) OASFs were incubated with different concentrations of IL-17 (0–10 ng/mL) for 24 h and miRNA expression was detected by qPCR. (**D**, **E**) OASFs were transfected with miR-4492 mimic or control mimic for 24 h, then stimulated with IL-17 (10 ng/mL) for a further 24 h. IL-18 production was assessed by qPCR and ELISA. (**F**, **G**) OASFs were pretreated with MER and ERK inhibitor or transfected with MEK, ERK, and control siRNAs, then stimulated with IL-17 (10 ng/mL) for a further 24 h. miR-4492 expression was evaluated by qPCR. Results are expressed as the means ± S.D. * p<0.05 compared with controls; # p<0.05 compared with the IL-17-treated group.

MiRNAs are small, noncoding RNAs that regulate cellular functions via post-transcriptional regulation and are involved in a variety of biological functions such as cell proliferation, differentiation, development, and programed cell death [40]. We therefore sought to determine which miRNAs regulate IL-18 expression. Searches of the online miRNA prediction databases miRWalk and miRDB identified 8 candidate miRNAs that potentially target IL-18 mRNA (Figure 6A). Validation of the expression of these miRNAs in OASFs treated with IL-17 revealed that of all miRNAs, the levels of miR-4492 were reduced the most after IL-17 exposure (Figure 6B, 6C). When we examined whether IL-18 expression was mediated by miR-4492, we observed that transfecting OASFs with miR-4492 mimic significantly downregulated IL-17-induced promotion of IL-18 (Figure 6D, 6E). We also observed that pretreating OASFs with MEK/ERK inhibitors or siRNAs dramatically reversed the downregulation of miR-4492 expression induced by IL-17 treatment (Figure 6F, 6G). These data suggest that IL-17 treatment increases levels of IL-18 expression in OASFs by reducing miR-4492 expression.

## DISCUSSION

IL-18 is an inflammatory cytokine that mediates many biological functions. Previous research has suggested a pivotal role for IL-18 in the pathogenesis of RA [41]. More recently, IL-18 has been proposed as a novel therapeutic target in RA disease [42]. Although several studies have revealed higher levels of IL-18 expression in OA patients than in normal healthy controls [12–15], the molecular mechanism underlying IL-18 production is unknown. We found in this study that IL-17 apparently promotes IL-18 expression in OASFs. Moreover, it appears that the MEK/ERK/miR-4492 axis is responsible for IL-17-induced stimulation of IL-18 expression. We suggest that IL-17, as a regulator of IL-18 production, may serve as a novel target in OA treatment.

The mitogen-activated protein kinase (MAPK) signaling pathway is activated by proinflammatory cytokines, or responsible for its production [43]. In this study, we found that IL-17 elicited MEK/ERK signaling and thus contributes to IL-18 production in OASFs. We also found that blockade of p38 and JNK pathway also inhibited IL-18 expression (Supplementary Figure 2). Of all MAPK components, the strongest and fastest to be activated by IL-17 stimulation is ERK [44]. It is also established that IL-17 participates in the pathogenesis of atopic dermatitis by activating ERK and p38 signaling [45]. Not only has MAPK been proposed as a key regulator in the production of proinflammatory cytokines that account for joint inflammation and destruction [46], but ERK has been found to mediate the production of IL-6, IL-12, IL-23 and tumor necrosis factor (TNF)-α [47, 48], which implicates a key role for ERK in inflammatory joint diseases via the promotion of proinflammatory cytokine release. Evidence from a rabbit model of OA indicates that inhibition of MAPK has the potential to slow the progression of OA, because the severity of OA lesions was reduced when ERK activation was blocked by a MEK1/2 inhibitor [49]. In addition, avocado soy unsaponifiables reportedly ameliorate cartilage degeneration and inflammation via ERK signaling [50].

The MAPK pathway has been implicated in the regulation of mRNA stability in cells stimulated by IL-17 [38]. TRBP phosphorylation by the Ras/MEK/ERK signaling cascade modulates miRNA biogenesis [51]. Notably, growth-promoting miR-17, miR-10a, and miR-92a are upregulated, whereas levels of the let-7 tumor suppressor miRNA family are downregulated in phospho-mimic TRBP-expressing Flp-In 293 cells, suggesting that TRBP phosphorylation simultaneously upregulates the pro-growth miRNA profile and downregulates levels of anti-growth miRNAs [51]. Recently, phosphorylation of TRBP was suggested to affect Dicer stability as a downstream consequence of altered TRBP stability, accounting for miRNA biogenesis [52]. Interestingly, our results showed that blocking the MEK/ERK signal dramatically restored miR-4492 expression after IL-17 stimulation, showing an overshooting phenomenon compared to the normal control. The prediction from a miRNA bioinformatics tool indicated that miR-4492 may target the 3’UTR of ERK (MAPK1) (Supplementary Figure 3). This finding suggests that the miR-4492/ERK feedback loop may be activated by IL-17, and the blockade of the MEK/ERK signal may result in an overshooting of miR-4492 expression due to an imbalance in the miR-4492/ERK feedback loop.

Interestingly, our results revealed the downregulation of miR-4492 in OASFs treated with IL-17. Little is known about miR-4492. Previous studies have indicated that miR-4492 participates in cancer progression through different mechanisms. In bladder cancer for instance, the miR-4492/ROMO1 axis controls the proliferation, migration, and invasion of cancer cells [53], while the miR-4492/FOXK1 axis has been implicated in the proliferation and invasion of colorectal cancer cells [54]. The long non-coding RNA (lncRNA) forkhead box D2 antisense 1 (FOXD2-AS1) reportedly enhances the proliferation, migration and invasion of ovarian cancer cells by targeting miR-4492, although the specific target of miR-4492 has not yet been verified [55]. Our study results indicate that miR-4492 may mediate immune responses by targeting IL-18, which also plays a key role in OA progression. Whether miR-4492 is involved in the pathogenesis of OA is worth future exploration.

## CONCLUSIONS

**Figure 7:**
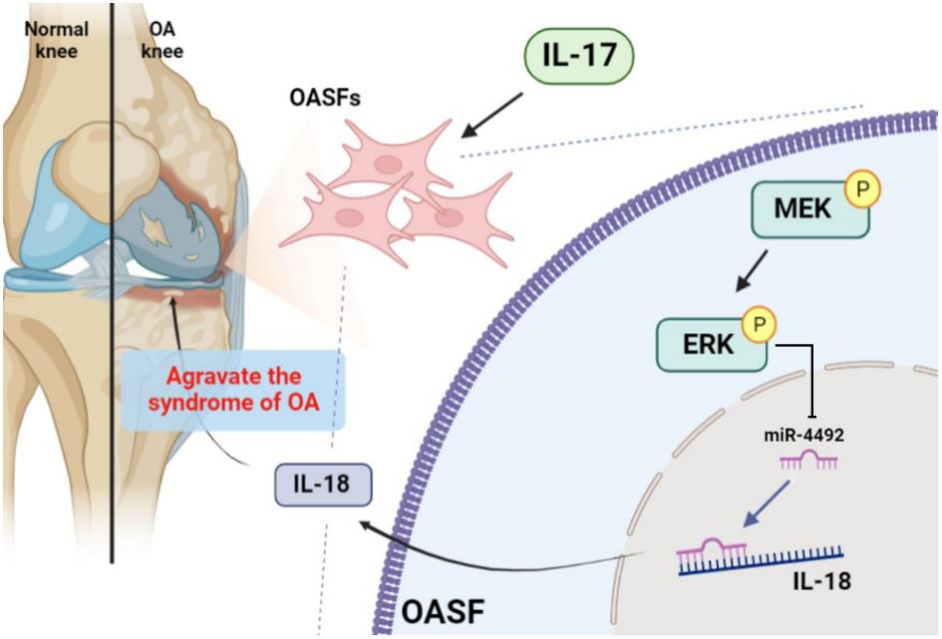
**Schematic diagram illustrates the process whereby IL-17 treatment promotes IL-18 production in OASFs.** IL-17 treatment upregulates levels of IL-18 expression in OASFs via MEK and ERK signaling and downregulates levels of miR-4492. IL-18 production elicits inflammatory responses during OA progression.

In conclusion, IL-17 promotes the production of IL-18 in OASFs via the MEK/ERK/miR-4492 axis (Figure 7). This study provides novel insights into the pathogenesis of OA and suggests a potential therapeutic target in OA treatment.

## Supplementary Material

Supplementary Figures
