# Supplementary material for: IL-17 promotes IL-18 production via the MEK/ERK/miR-4492 axis in osteoarthritis synovial fibroblasts
Source: Aging (Albany NY). 2024 Jan 22;16(2):1829–44. doi: 10.18632/aging.205462 (PMC10866453; doi:10.18632/aging.205462)
Supplement: Supplementary Figures [file aging-16-205462-s001.pdf]

SUPPLEMENTARY FIGURES

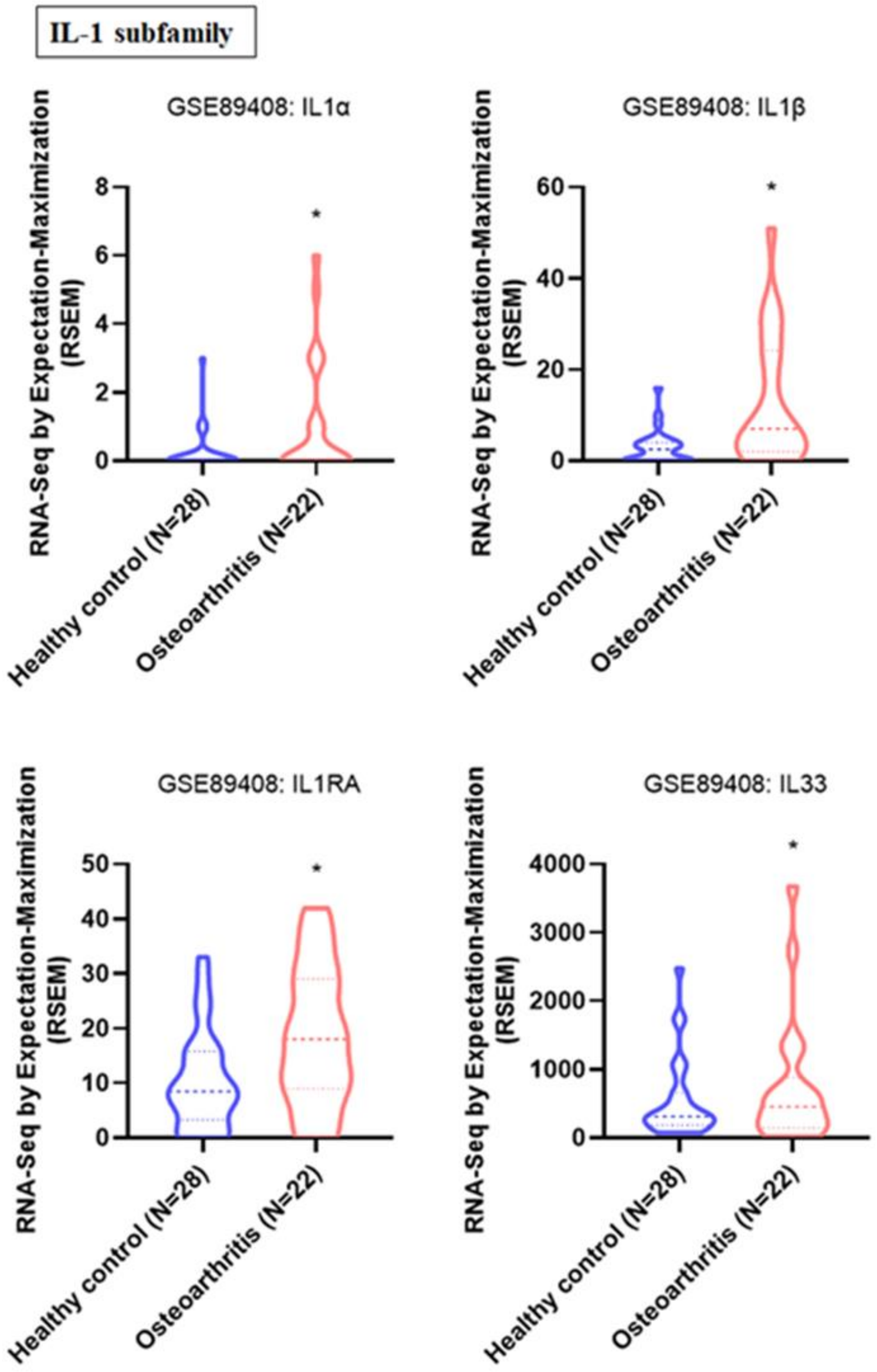

Supplementary Figure 1. IL-1 family members and its receptor are up-regulated in specimens of OA patients. The GEO dataset (GSE890408) contained mRNA expression pattern from the synovial tissues of OA patients and normal donors were used to analyze IL-1α, IL-1β, IL-33 and IL-RA expressions. Results are expressed as the means ± S.D. * $p<0.05$ compared with controls.

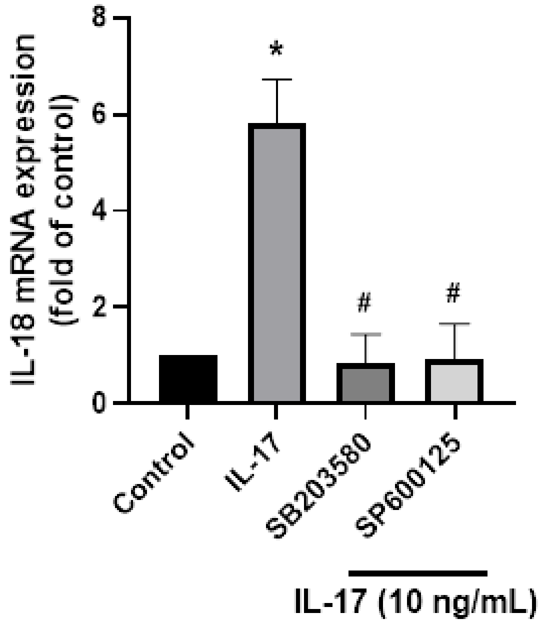

Supplementary Figure 2. IL-17-induced promotion of IL-18 production in OASFs requires p38 and JNK signaling. OASFs were pretreated with p38 and JNK inhibitors SB203580 (5 μM) or SP600125 (5 μM) for 1 h, then incubated with IL-17 (10 ng/mL) for 24 h. IL-18 expression was determined by qPCR. Results are expressed as the means ± S.D. * p<0.05 compared with controls; # p<0.05 compared with the IL-17-treated group.

	Ensembl Gene Id	miRNA name	miTG score	Also Predicted
1	ENSG00000100030 (MAPK1)	hsa-miR-4492	0.885935706270861	

Gene details
miRNA details
pubMed links: miRNA | gene | both
UCSC graphic

Region	Binding Type	Transcript position	Score	Conservation
UTR3	7mer	9659-9675	0.015976824446552	5
UTR3	6mer	9542-9548	0.00296085739835613	2
UTR3	7mer	9282-9306	0.012355223162973	4
UTR3	6mer	7818-7835	0.00163176721363082	2
UTR3	8mer	7690-7711	0.00147332894034286	2
UTR3	6mer	695-714	0.00271090948120946	2
UTR3	6mer	309-337	0.0032522213901444	2
UTR3	6mer	92-110	0.00384781608633246	1

Supplementary Figure 3. Prediction result of mir-4492 binding to ERK (MAPK1) 3'UTR using the DIANA-microT-CDS tool. The online database for miRNA target prediction (DIANA-microT-CDS) was utilized to predict miR-4492, which is a potential target for ERK mRNA.
